# Comparative study of eGFR in cancer and non-cancer individuals: a multicenter analysis

**DOI:** 10.3389/fmed.2025.1642162

**Published:** 2025-12-04

**Authors:** Baokui Ye, Xin Luo, Yunhan Luo, Zhaohui Zhou, Biao Jiang, Longbin Xiong, Yulu Peng, Xiangpeng Zou, Yixin Huang, Yisong Lin, Lihao Zhang, Xiaofeng Yang, Pei Dong, Yuying Liu, Yue Yan, Xiaohua Liu, Jiawei Xie, Yulong Xiao, Bokang Cui, Song Wang, Jinchang Lu, Zhiting He, Huiming Liu, Jing Li, Zhen Li, Wei Lu, Yancen Li, Haiying Liu, Tao Jia, Lizhi Niu, Fangjian Zhou, Chunping Yu, Zhiling Zhang

**Affiliations:** 1 Department of Intensive Care Unit, Sun Yat-sen University Cancer Center, Guangzhou, China; 2 State Key Laboratory of Oncology in South China, Guangdong Provincial Clinical Research Center for Cancer, Sun Yat-sen University Cancer Center, Guangzhou, China; 3 Department of Urology, Sun Yat-sen University Cancer Center, Guangzhou, China; 4 Department of Urology, Minimally Invasive Surgery Center, The First Affiliated Hospital of Guangzhou Medical University, Guangzhou, China; 5 Screening Center for Cancer Prevention, Sun Yat-sen University Cancer Center, Guangzhou, China; 6 Department of Clinical Laboratory, Sun Yat-Sen University Cancer Center, Guangzhou, China; 7 Department of Thoracic Surgery, Sun Yat-Sen University Cancer Center, Guangzhou, China; 8 Department of Gastric Surgery, Sun Yat-Sen University Cancer Center, Guangzhou, China; 9 Department of Hepatobiliary Oncology, Sun Yat-Sen University Cancer Center, Guangzhou, China; 10 Department of Colorectal Surgery, Sun Yat-Sen University Cancer Center, Guangzhou, China; 11 Department of Musculoskeletal Oncology, Sun Yat-Sen University Cancer Center, Guangzhou, China; 12 Department of Breast Surgery, Sun Yat-Sen University Cancer Center, Guangzhou, China; 13 Department of Medical Imaging, Sun Yat-Sen University Cancer Center, Guangzhou, China; 14 Guangzhou Institute of Cancer Research, The Affiliated Cancer Hospital, Guangzhou Medical University, Guangzhou, China; 15 Department of Urology, Hunan Provincial People's Hospital, The First Affiliated Hospital of Hunan Normal University, Changsha, China; 16 Department of Preventive Medicine, Guangzhou Concord Cancer Center, Guangzhou, Guangdong, China; 17 Department of Urology, The First People's Hospital of Chenzhou, Chenzhou, China; 18 Medical Examination Center, Shenzhen Baoan Shiyan People's Hospital, Shenzhen, Guangdong, China; 19 The People's Hospital of Zheng'an, Zunyi, China; 20 Biological Treatment Center, Fuda Cancer Hospital, Jinan University, Guangzhou, China; 21 Department of Oncology, Fuda Cancer Hospital, Jinan University, Guangzhou, China

**Keywords:** cancer, eGFR, oncology, kidney volumes, multicenter study

## Abstract

**Background:**

Cancer is the second leading cause of death worldwide, and renal impairment frequently occurs during its course and treatment due to nephrotoxic chemotherapy and contrast exposure. Understanding baseline estimated glomerular filtration rate (eGFR) and its longitudinal changes in cancer patients is essential for effective kidney function management.

**Methods:**

This multicenter study included 24,478 treatment-naïve cancer patients admitted to three cancer centers in 2018 and 61,883 non-cancer individuals undergoing health screening in the same year, with follow-up until 2023. eGFR was calculated using the CKD-EPI equation. Baseline eGFR and subsequent changes were compared between groups after stratification by age, sex, and comorbidity.

**Results:**

Across all age groups, treatment-naïve cancer patients showed higher baseline eGFR values compared with non-cancer individuals of the same stratum (all *p* < 0.05). The median kidney volume was also larger in cancer patients (280 cm^3^ vs. 271 cm^3^, *p* < 0.05). During the 5-year follow-up, cancer patients demonstrated a greater annual decline in eGFR than non-cancer individuals (all *p* < 0.01).

**Conclusion:**

Treatment-naïve cancer patients exhibited higher baseline eGFR but a more pronounced subsequent decline compared with non-cancer individuals. These findings reflect observed associations rather than causal effects.

## Introduction

Despite the rising global incidence of cancer, which is the second leading cause of death worldwide, advancements in anti-cancer treatment have gradually improved the five-year survival rate (1). Against this backdrop, the life expectancy of cancer patients has significantly increased, highlighting the importance of improving their quality of life. Renal function decline is common in patients undergoing cancer treatment and is strongly linked to tumor-related morbidity and mortality (2). Therefore, monitoring renal function is crucial for enhancing cancer treatment outcomes and ensuring patients’ quality of life (3, 4).

Numerous studies have highlighted the importance of monitoring renal function in cancer patients. Hong et al. (5) found that baseline renal impairment is an independent prognostic marker associated with inferior overall survival in cancer patients. Moreover, renal dysfunction is more frequent in cancer patients undergoing treatment, with one large cohort study reporting that 25.8% of cancer patients developed acute kidney injury (AKI) during follow-up (2). In another multicenter cohort, over half of cancer patients were found to have renal insufficiency (6). Notably, most of these studies have primarily focused on patients who had already undergone anticancer treatment, which itself can affect renal function. Furthermore, while some studies had baseline renal function data of cancer patients without treatment, they did not compare it with non-cancer individuals (5, 7). In summary, there is a lack of studies comparing baseline renal function between untreated cancer patients and non-cancer controls, as well as evaluating post-treatment renal function changes relative to non-cancer individuals.

When comparing these groups, demographic and clinical characteristics—such as age, general health status, and comorbidities (e.g., diabetes and hypertension)—may confound the relationship between cancer and renal function. Careful consideration of these variables is essential for accurate interpretation of any observed differences.

To address this knowledge gap, we conducted a multicenter, descriptive, and primarily cross-sectional study comparing baseline eGFR and five-year renal function trajectories between treatment-naïve cancer patients and non-cancer individuals. Baseline comparisons were stratified by age, sex, and comorbidities to minimize confounding. We observed that cancer patients had higher baseline eGFR but a faster decline over time. Rather than representing “fragile eGFR,” these findings may indicate a transient, cancer-related hyperfiltration pattern that predisposes patients to accelerated renal function loss after treatment. This study is exploratory and hypothesis-generating, aiming to provide population-level insight into cancer-related renal alterations.

## Materials and methods

### Study design and participants

This study was designed as a multicenter, descriptive, and primarily cross-sectional analysis with a longitudinal follow-up component. It was primarily conducted at our center, with data collected from our institution and other collaborating hospitals and clinics (details in Table 1). Baseline comparisons between cancer and non-cancer participants were performed cross-sectionally, while changes in renal function over time (2018–2023) were analyzed descriptively in the subset of participants with available follow-up data. The primary purpose of the study was exploratory and hypothesis-generating rather than causal inference.

**Table 1:** Baseline characteristics of participants from all centers.

Variable	Cancer subjects			Non-cancer subjects					
	Center 1 (our center)	Center 2	Center 3	Center 4 (screening center in our center)	Center 5	Center 6	Center 7	Center 8	Center 9
Participants (*n*)	22,436	1,220	822	20,597	4,620	1828	9,972	19,752	5,114
Age									
Mean	52.3	56.1	55.9	40.5	42.3	43.1	45.8	42.2	36.6
Median	52.8	57.0	55.6	38.7	42.0	41.0	46.0	39.0	32.0
Woman (%)	10,628 (47.4)	530 (43.3)	347 (42.2)	12,278 (59.6)	1,543 (33.3)	1,064 (58.2)	98 (45.7)	10,799 (54.7)	2,317 (45.3)
BMI									
Mean	22.6	22.3	22	22.8	24.3	23.5	/	/	/
Median	22.5	22.1	21.8	22.5	24.3	23.0	/	/	/
Hypertension	2,582 (11.5)	192 (15.7)	141 (17.2)	1826 (8.9)	876 (19.0)	214 (11.7)	/	/	/
Diabetes	1767 (7.9)	204 (16.7)	141 (17.2)	848 (4.1)	212 (4.6)	78 (4.3)	/	/	/
eGFR, *n* (%)									
>120 mL/min per 1.73 m^2^	2,560 (11.4)	91 (7.4)	107 (13.2)	3,678 (17.9)	1,014 (21.9)	341 (18.7)	1,457 (14.6)	4,370 (22.1)	1700 (33.2)
90–120 mL/min per 1.73 m^2^	16,249 (72.4)	913 (74.8)	581 (70.7)	14,471 (70.3)	2,966 (64.2)	1,241 (67.9)	6,860 (68.8)	12,813 (64.9)	3,032 (59.3)
60–90 mL/min per 1.73 m^2^	3,169 (14.1)	193 (15.8)	103 (12.5)	2,348 (11.4)	576 (12.5)	236 (12.9)	1,536 (15.4)	2,402 (12.2)	365 (7.1)
<60 mL/min per 1.73 m^2^	458 (2.0)	23 (1.9)	31 (3.8)	100 (0.5)	64 (1.4)	10 (0.5)	119 (1.2)	167 (0.8)	17 (0.3)
TNM stage									
I	5,724 (25.5)	148 (12.1)	39 (4.7)	/	/	/	/	/	/
II	4,417 (19.7)	209 (17.1)	46 (5.6)	/	/	/	/	/	/
III	6,671 (29.7)	314 (25.7)	171 (20.7)	/	/	/	/	/	/
IV	5,624 (25.1)	549 (45.0)	570 (69.0)	/	/	/	/	/	/

BMI, body mass index; eGFR, estimated Glomerular Filtration Rate.

A total of 24,478 cancer patients and 61,883 non-cancer individuals were included. All cancer patients were newly diagnosed in 2018, with follow-up data extending until 2023. Inclusion criteria for cancer patients were individuals aged >18 years with a pathological diagnosis of malignant solid tumors, excluding those with kidney tumors, upper urinary tract urothelial carcinoma, hematologic malignancies, or a history of nephrectomy. Non-cancer individuals were selected based on age, sex, and geographic matching to the cancer cohort to ensure baseline comparability.

The study was approved by the Institutional Review Board of our center and all collaborating hospitals and clinics, and conducted in accordance with the ethical standards of the Declaration of Helsinki. All data were anonymized prior to analysis to ensure patient confidentiality.

### Data sources and collection

Data for the target population were extracted from the electronic medical records of our center and collaborating hospitals, spanning January 2018 to December 2023. The extracted data included patient demographics, clinical characteristics, renal function parameters (e.g., serum creatinine), treatment details, and imaging results. Each diagnosis was verified through a comprehensive review of medical records.

### Variables and measurements

The primary variables in this study included the estimated glomerular filtration rate (eGFR), treatment information for renal tumor patients, and comorbidity status. eGFR was calculated from serum creatinine levels using the CKD-EPI equation, which is considered one of the most accurate formulas for estimating renal function in cancer patients (8, 9). Renal parenchymal volume was measured through 3D reconstruction based on the portal venous phase of contrast-enhanced CT, performed by an experienced urologic radiologist using MIMICS software (Materialise NV, Leuven, Belgium). All measurements performed by a single experienced radiologist. The radiologist was blinded to patient group status to minimize bias.

### Statistical analysis

Data were analyzed using descriptive statistics for baseline characteristics, with group differences assessed using paired t-tests for continuous variables and Chi-square tests for categorical variables. To adjust for confounding factors, propensity score matching was performed using covariates including age, sex, and comorbidities. Differences in kidney function and renal volume between groups were assessed using linear regression models adjusted for matching covariates. Mixed-effects models were employed to account for clustering within centers. Statistical significance was set at *p* < 0.05.

## Results

### Baseline characteristics

The baseline characteristics of the study population are summarized in Table 1. A total of 86,365 participants were enrolled in the study, comprising 24,478 cancer patients from three cancer centers and 61,883 non-cancer individuals from six different hospitals and health examination centers.

Among the 24,478 cancer patients from the three cancer centers, the mean age was 53.3, 56.1, 56.9 years old, respectively. The proportion of female patients varied from 42.2 to 47.4% across the three centers. The prevalence of hypertension among cancer patients ranged from 11.5 to 17.2%, while the prevalence of diabetes ranged from 7.9 to 17.2%. Approximately 72% of patients having an eGFR between 90 and 120 mL/min/1.73 m^2^, while only a small proportion (2.0–3.8%) had an eGFR <60 mL/min/1.73 m^2^. In terms of cancer stage, the proportion of stage I to IV patients being varied among three cancer centers.

For non-cancer participants across all centers, the mean age ranged from 36.6 to 45.8 years old, with the proportion of women varying between 33.3 and 58.2% across centers. The prevalence of hypertension ranged from 8.9 to 19.0%, while diabetes ranged from 4.1 to 4.6%. A majority of non-cancer participants (approximately 70%) had an eGFR between 90 and 120 mL/min/1.73 m^2^, while a smaller proportion (17.9%) had an eGFR >120 mL/min/1.73 m^2^. Less than 1.4% of non-cancer participants had an eGFR <60 mL/min/1.73 m^2^, indicating generally normal renal function in the non-cancer population.

### eGFR comparison between cancer and non-cancer participants across multiple centers

Given the differences in baseline characteristics between the cancer and non-cancer groups—such as age, sex distribution, and prevalence of comorbidities—and the potential influence of these factors on eGFR levels, eGFR comparisons were initially stratified by age. Within each age stratum, further subgroup analyses were conducted based on sex and the presence of comorbidities to provide a comprehensive understanding of renal function differences between cancer and non-cancer patients.

**Figure 1:**
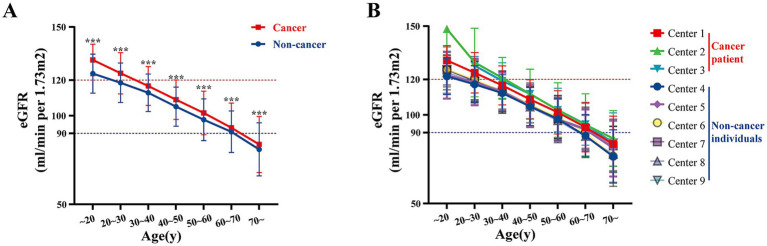
Multi-center age-stratified analysis of eGFR in cancer vs. non-cancer groups. **(A)** The overall distribution of eGFR levels across different age groups for multiple centers, categorized by non-cancer individuals and cancer patients. **(B)** The eGFR levels for each individual center, stratified by age groups, separately displaying the values for non-cancer individuals and cancer patients. Centers 1–3 represent data of cancer patients and centers 4–9 represent data of non-cancer individuals. ***p < 0.001.

The overall comparison of eGFR levels between cancer and non-cancer patients is illustrated in Figure 1. Across all centers, untreated cancer patients demonstrated a trend of having higher baseline eGFR compared with non-cancer group. Specifically, the mean eGFR was consistently elevated in cancer patients across different age groups when compared to non-cancer subjects (all *p* < 0.05) (Figure 1A). Notably, this trend was observed in each of the participating institutions (Figure 1B).

**Figure 2:**
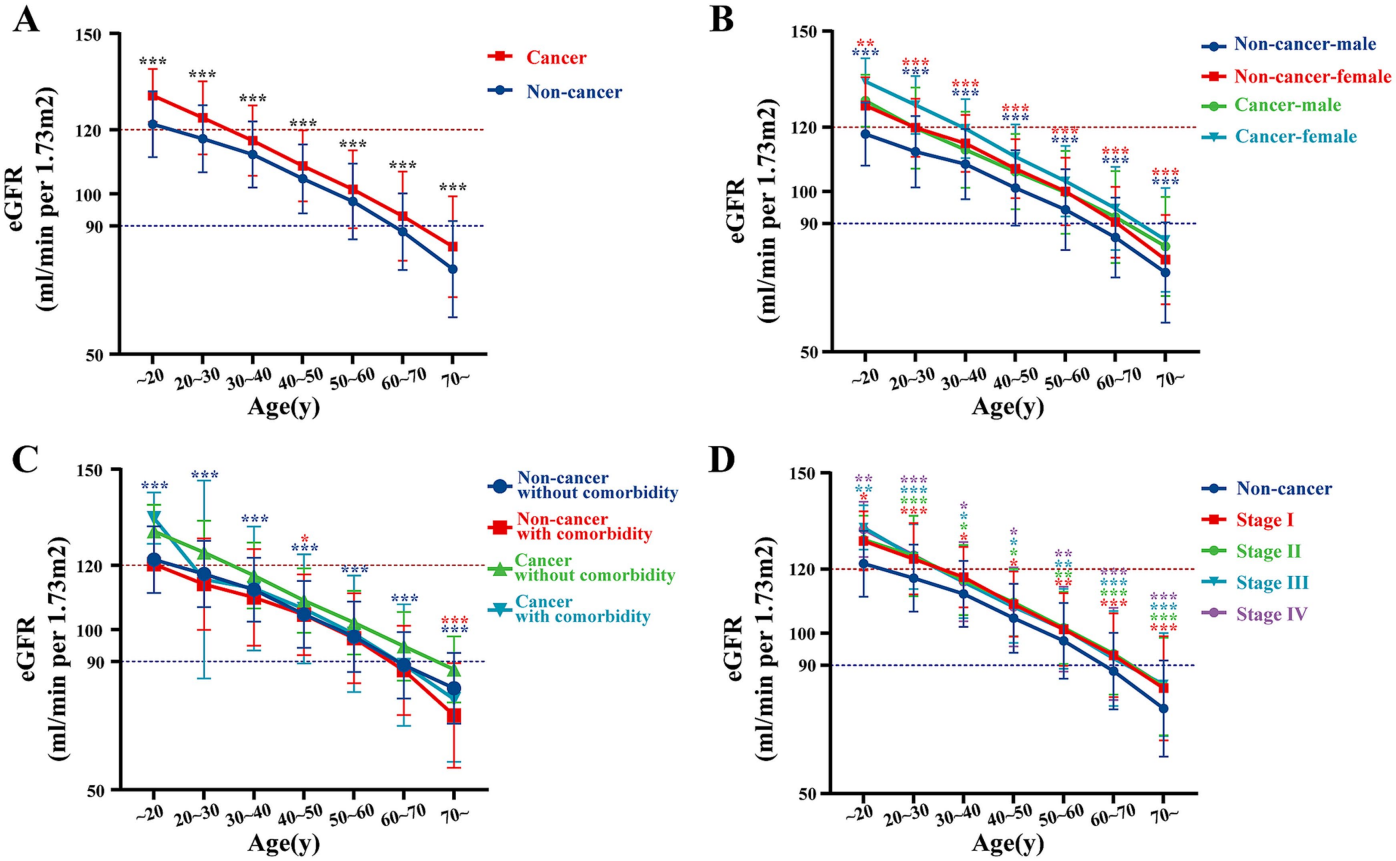
Comparison of eGFR, renal parenchymal volume, and annual GFR decline between cancer and non-cancer groups at our center. **(A)** eGFR in cancer vs. non-cancer groups by age at our center. **(B–D)** eGFR in cancer vs. non-cancer groups stratified by different factors at our center: **(B)** by age and gender, **(C)** by comorbidity, and **(D)** by cancer stage.

To investigate the factors that may influence eGFR levels, we conducted a detailed analysis on patients from our center. The line chart illustrates that baseline eGFR in cancer patients was higher than in non-cancer individuals across all age strata (Figure 2A). This observation persisted when stratified by sex, comorbidities, and cancer stage (Figures 2B–D). Specifically, both male and female cancer patients exhibited higher baseline eGFR compared to their non-cancer counterparts (all *p* < 0.05). Similar trends were noted in cancer patients with or without comorbidities (all p < 0.05), as well as in patients across different cancer stages (all *p* < 0.05), with no significant differences observed between different cancer stages (all >0.05). These findings were also validated using data from center 2 and center 3 (Supplementary Figure S1). These results suggest that untreated cancer patients may exhibit relatively higher eGFR at baseline.

### Multivariable analysis of factors associated with eGFR

**Figure 3:**
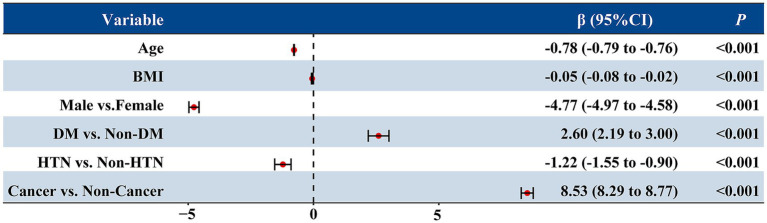
Multivariable linear regression analysis of factors associated with estimated glomerular filtration rate (eGFR). The forest plot displays the *β* coefficients and 95% confidence intervals (CIs) for each variable included in the model. Age, sex, BMI, diabetes, hypertension, and cancer status were entered as independent variables. Negative *β* values indicate lower eGFR, while positive values indicate higher eGFR. All *p* values were <0.001.

To identify independent determinants of renal function, a multivariable linear regression analysis was performed including age, sex, body mass index (BMI), diabetes, hypertension, and cancer status (Figure 3). The model explained approximately 46% of the variability in eGFR (adjusted *R*^2^ = 0.462, *p* < 0.001).

Age was strongly and inversely associated with eGFR (*β* = −0.78, *p* < 0.001), indicating an average decline of 0.78 mL/min/1.73 m^2^ per year of age. Male sex was associated with a 4.8 mL/min/1.73 m^2^ lower eGFR compared with females (*p* < 0.001), and higher BMI was also modestly associated with lower eGFR (*β* = −0.05, *p* = 0.001). Hypertension was linked to a modest decrease in eGFR (*β* = −1.22, *p* < 0.001), whereas diabetes showed a slight positive association (*β* = 2.60, *p* < 0.001), likely reflecting hyperfiltration at baseline.

Notably, after adjusting for these covariates, cancer status remained an independent predictor of higher eGFR (*β* = 8.53, *p* < 0.001), suggesting that the elevated eGFR observed in cancer patients cannot be fully explained by demographic or metabolic factors.

### Propensity score matching analysis

To further eliminate potential confounding effects from baseline differences, a 1:1 nearest-neighbor PSM was conducted using age, sex, BMI, diabetes, and hypertension as covariates (caliper = 0.05).

After matching, 26,046 participants were included (13,023 cancer and 13,023 non-cancer individuals) and the baseline characteristics between the two groups were well balanced (Table 2, Supplementary Table S1). The mean age was comparable between cancer and non-cancer participants (49.75 ± 11.0 vs. 49.38 ± 10.9 years, *p* = 0.007, SMD = 0.033). The distributions of sex, BMI, diabetes, and hypertension were also similar between groups (*p* > 0.05, all SMDs <0.05).

**Table 2:** Baseline characteristics after propensity score matching.

Variable	Overall (*n* = 26,046)	Non-cancer (*n* = 13,023)	Cancer (*n* = 13,023)	*p*	SMD
Sex (%)					
Woman	13,978 (53.7%)	7,022 (53.9%)	6,956 (53.4%)	0.419	0.01
Man	12,068 (46.3%)	6,001 (46.1%)	6,067 (46.6%)		
Age (mean ± SD)	49.57 (10.95)	49.38 (±10.87)	49.75 (±11.04)	0.007	0.033
BMI (mean ± SD)	23.00 (3.23)	23.06 (±2.93)	22.95 (±3.50)	0.011	0.032
Diabetes (DM) (%)					
No	24,402 (93.7%)	12,210 (93.8%)	12,192 (93.6%)	0.665	0.006
Yes	1,644 (6.3%)	813 (6.2%)	831 (6.4%)		
Hypertension (HTN) (%)					
No	22,902 (87.9%)	11,469 (88.1%)	11,433 (87.8%)	0.506	0.008
Yes	3,144 (12.1%)	1,554 (11.9%)	1,590 (12.2%)		

SD, standard deviation; BMI, body mass index; SMD, standard mean difference.

**Figure 4:**
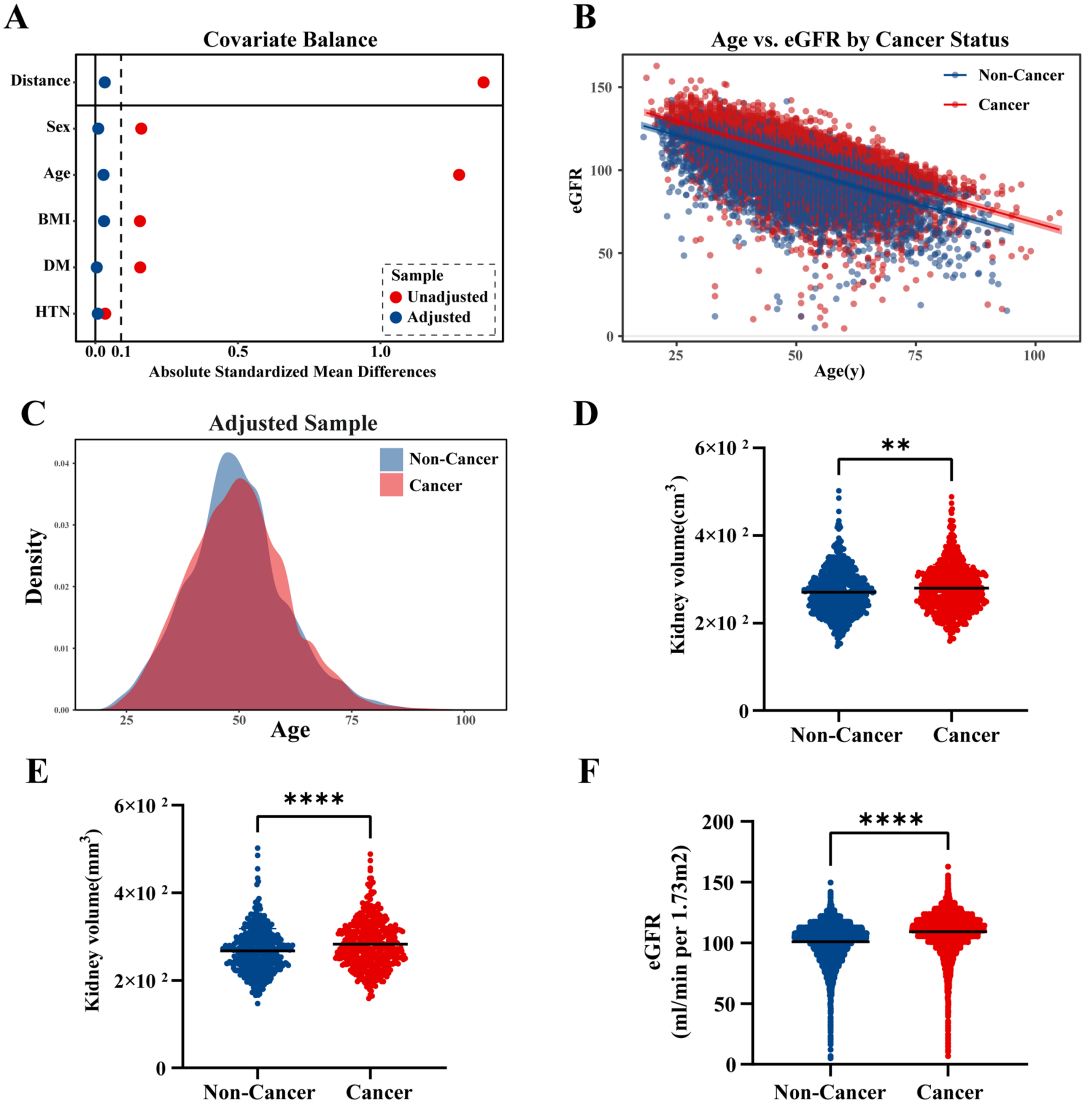
**(A)** Covariate balance before and after matching, as shown by absolute SMDs for each covariate (distance, sex, age, BMI, diabetes, hypertension). The red dots represent the unadjusted values, while the blue dots represent the adjusted values post-PSM. **(B)** The relationship between age and eGFR, stratified by cancer status, showing that cancer patients (red) exhibit a steeper decline in eGFR with increasing age compared to non-cancer patients (blue). **(C)** Age distribution in the adjusted sample, with cancer patients (red) and non-cancer patients (blue) showing similar distributions after matching. **(D)** Kidney volume comparison before PSM. The figure shows a significant difference in kidney volume between non-cancer patients (blue) and cancer patients (red) prior to matching (271 ± 52 vs. 280 ± 54, *p* < 0.01). **(E)** Kidney volume comparison after PSM. After matching, kidney volume was still significantly smaller in non-cancer patients compared to cancer patients (267 ± 51 vs. 283 ± 55, *p* < 0.0001). **(F)** Comparison of eGFR between cancer and non-cancer patients after PSM. Non-cancer patients (blue) exhibited significantly lower eGFR compared to cancer patients (red) post-matching (100.6 ± 15.3 vs. 109.9 ± 13.8, *p* < 0.0001).

All covariates achieved satisfactory balance after matching, with all absolute SMD below 0.05, indicating that sex, BMI, diabetes, and hypertension were well controlled. Among these variables, age still exhibited a minimal but non-zero residual difference (SMD = 0.033, Figure 4A); therefore, an additional age-adjusted regression analysis was performed to account for this slight imbalance.

In the matched cohort, multivariable linear regression adjusting for age demonstrated that cancer patients continued to have a significantly higher baseline eGFR compared with non-cancer individuals (*β* = 8.56, 95% CI 8.29–8.84, *p* < 0.001), whereas age remained independently associated with lower eGFR (*β* = −0.82, 95% CI –0.83 to −0.81, *p* < 0.001) (Supplementary Table S2, Figures 4B,C).

These findings confirm that the higher baseline eGFR observed in cancer patients persisted even after rigorous adjustment for demographic and metabolic covariates, supporting the presence of a cancer-related renal hyperfiltration pattern independent of age and comorbidities.

### Renal parenchyma volume enlargement and accelerated eGFR decline in cancer patients

To further investigate the cause of the higher baseline eGFR observed in cancer patients, we measured the bilateral kidney volume of both cancer and non-cancer individuals after performing PSM. Following PSM, 862 participants were included (431 cancer patients and 431 non-cancer individuals), and their baseline characteristics were well balanced in terms of sex, age, BMI, diabetes, and hypertension (Table 3, Supplementary Table S3).

**Table 3:** Baseline characteristics after propensity score matching.

Variable	Overall (*n* = 862)	Non-cancer (*n* = 431)	Cancer (*n* = 431)	*p*	SMD
Sex (%)					
Woman	0.61 (0.49)	0.62 (0.49)	0.60 (0.49)	0.577	0.038
Man	0.39 (0.49)	0.38 (0.49)	0.40 (0.49)		
Age (mean ± SD)	47.75 (±11.20)	47.77 (±12.32)	47.74 (±9.98)	0.97	0.003
BMI (mean ± SD)	23.52 (±2.99)	23.63 (±2.85)	23.42 (±3.13)	0.317	0.068
Diabetes (DM) (%)					
No	0.95 (0.22)	0.94 (0.23)	0.95 (0.21)	0.536	0.042
Yes	0.05 (0.22)	0.06 (0.23)	0.05 (0.21)		
Hypertension, HTN (%)					
No	0.92 (0.26)	0.92 (0.28)	0.93 (0.25)	0.367	0.061
Yes	0.08 (0.26)	0.08 (0.28)	0.07 (0.25)		
Kidney volume (mean ± SD, cm^3^)	275 (±53)	267 (±51)	283 (±55)	<0.001	0.296

SD, standard deviation; BMI, body mass index; SMD, standard mean difference.

Despite the balanced baseline characteristics, a significant difference in kidney volume remained between the two groups. Cancer patients exhibited a larger bilateral kidney volume and higher eGFR compared to non-cancer patients (283 ± 55 cm³ vs. 267 ± 51 cm³, *p* < 0.001, SMD = 0.296) (Table 3, Figures 4D–F). This suggests that the elevated baseline eGFR in cancer patients may be attributable to compensatory renal hypertrophy associated with cancer pathology, even after adjusting for other demographic and metabolic factors.

These findings highlight the potential role of renal parenchyma enlargement in the higher eGFR observed in cancer patients, supporting the hypothesis that renal hyperfiltration in cancer is likely a result of adaptive changes in kidney volume.

### Long-term renal function decline in cancer patients

**Figure 5:**
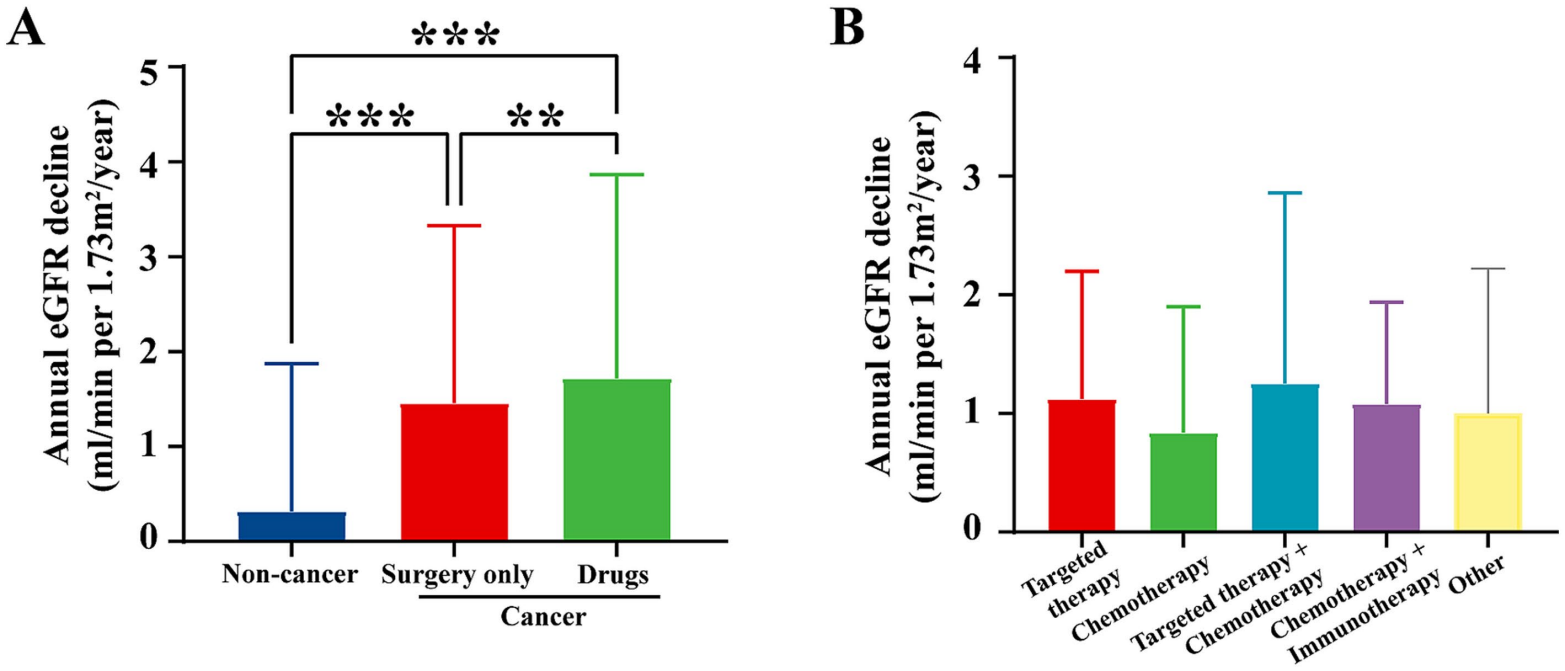
Annual eGFR decline by treatment regimen from 2018 to 2023. **(A)** Annual eGFR decline by treatment regimen at our center from 2018 to 2023, compared to non-cancer individuals. Data for non-cancer individuals are exclusively from the screening center at our institution. The data show that cancer patients undergoing surgery or drug treatments exhibited significantly higher annual eGFR decline compared to non-cancer patients. *p* < 0.05, *p* < 0.01, and *p < 0.001. **(B)** Annual eGFR decline by treatment regimen (targeted therapy, chemotherapy, targeted therapy + chemotherapy, chemotherapy + immunotherapy, and other therapies). The graph shows the annual eGFR decline for cancer patients with various treatment regimens. Targeted therapy resulted in the least decline in renal function, while chemotherapy-based regimens exhibited a faster decline in eGFR.

Despite the initial elevation in eGFR observed in cancer patients in 2018, a notable difference emerged when renal function was reassessed in 2023. The annualized decline in eGFR, measured as the decrease in renal function from 2018 to 2023, was significantly faster in cancer patients compared to non-cancer individuals (Figure 5A).

Cancer patients who underwent surgical intervention or chemotherapy experienced a notably steeper decline in eGFR. This was especially true for patients treated with nephrotoxic agents, including certain chemotherapy drugs, which are known to cause kidney damage (Figure 5B). These findings highlight that the earlier increase in eGFR may have been a compensatory response to malignancy, rather than an indication of sustained renal health.

The use of nephrotoxic medications during cancer treatment may contribute to the accelerated renal function decline, suggesting that the initial renal hyperfiltration observed in cancer patients might be transient and not indicative of long-term kidney preservation. This emphasizes the importance of close monitoring of renal function in cancer patients, especially those undergoing chemotherapy or other treatments known to affect kidney health.

## Discussion

With increasing cancer survival rates, improving quality of life has become a critical focus (1). Preserving renal function is vital for improving the quality of life in cancer patients (2). A large-scale study in China, surveying over 7 million patients, reported that the incidence of AKI, defined as a 50% or greater increase in baseline serum creatinine, ranged from 14 to 20%, depending on hospital type (community vs. academic) (10). Additionally, other studies have found that the overall one-year incidence of AKI in cancer patients ranges from 11 to 20%, with individuals diagnosed with hematological cancers being at higher risk (2, 11). Aside from AKI, the prevalence of chronic kidney disease (CKD) in cancer patients has also been reported, with rates of 12.02% in France, 16.1% in Austria, and 13.4% in a Romanian study (6, 12, 13). However, these previous studies focus on patients post-treatment, overlooking untreated cancer patients and rarely comparing them to non-cancer individuals. To fill this gap in understanding of cancer’s direct impact on renal function, we conducted a multicenter, cross-sectional study comparing baseline eGFR and eGFR changes between 24,482 treatment-naïve cancer patients and 61,883 non-cancer individuals.

Our study found that treatment-naïve cancer patients had higher eGFR levels across all age groups compared with non-cancer individuals, even after adjusting for gender and comorbidities. Additionally, cancer patients had a larger median kidney volume. However, during the 5-year follow-up after surgery, medication, and other cancer treatments, cancer patients experienced a significantly greater annual decline in eGFR compared to non-cancer individuals.

In our study, pre-treatment eGFR levels in cancer patients were significantly higher than those in non-cancer individuals, likely due to physiological or metabolic changes specific to cancer patients. First, the increased metabolic rate or inflammatory state caused by certain cancers can lead to elevated renal blood flow, resulting in higher eGFR levels. For instance, prostaglandin E2 (PGE2) is the most abundant prostaglandin found in various human malignancies (14), and it acts as a potent vasodilator in renal vessels (15), significantly increasing renal blood flow and contributing to renal hyperperfusion. This mechanism may also partly explain the larger kidney volumes observed in cancer patients compared to non-cancer individuals. Second, malignant tumors release nitric oxide (NO) reported in many studies (16–18), which can alter glomerular hemodynamics and increase glomerular filtration rate (GFR). Furthermore, the elevated metabolic activity of malignancies leads to increased production of metabolic waste products (e.g., urea), which in turn raises osmotic pressure (19). The increase in osmotic pressure stimulates the kidneys to enhance filtration in order to expel excess solutes, thereby maintaining homeostasis. However, all these conjecture require further large-scale clinical verification.

During the subsequent five-year follow-up, the annual decline in eGFR was significantly greater in patients who underwent cancer treatments compared to non-cancer individuals. This suggests that cancer treatments have a notable negative impact on renal function. Various cancer therapies, including chemotherapy, immunotherapy, and targeted therapy, are known to have varying degrees of nephrotoxicity. Additionally, common complications during cancer treatment, such as dehydration, hypovolemia, or systemic inflammation, may accelerate the decline in renal function. Notably, despite some patients undergoing only surgical treatment, we observed a more rapid decline in renal function compared to non-cancer individuals. This could be attributed to compensatory hyperfiltration observed in cancer patients, which may initially assist in eliminating accumulated metabolic waste. However, prolonged hyperfiltration can lead to glomerular damage, ultimately contributing to the accelerated decline in renal function. Furthermore, malignancies themselves can directly damage renal tissues through multiple mechanisms. Tumors may induce renal injury through paraneoplastic syndromes such as immune-mediated responses (20). These processes can cause persistent and progressive renal injury, exacerbating the decline in renal function independently of therapeutic interventions.

These findings have significant clinical implications for the management of cancer patients. Although cancer patients may present with higher eGFR at diagnosis, their renal function is highly susceptible to decline during subsequent treatment. This highlights the need for close monitoring of renal function in cancer patients, particularly during and after treatment, to minimize nephrotoxicity and optimize overall outcomes. Regular assessment of renal function, including eGFR, should be an integral part of cancer management, especially when making decisions regarding surgery, imaging, and chemotherapeutic dosing, to protect renal health and improve prognosis.

The current study has some limitations. First, the cross-sectional design does not allow us to establish a causal relationship between cancer status and changes in renal function. Longitudinal studies are needed to better understand the dynamic changes in eGFR over time and to determine the direct impact of specific cancer treatments on renal function. Second, potential confounding factors, such as tumor type, treatment modality, and lifestyle factors, may not have been fully controlled, which could have influenced our findings. Additionally, the study included data from only three cancer centers, potentially limiting the generalizability of the results. Second, potential selection bias in the control group cannot be completely excluded. Although non-cancer individuals were selected from the same health screening database using strict inclusion criteria, they might differ from cancer patients in unmeasured confounders such as health-seeking behaviors or subclinical conditions. To minimize this bias, we applied PSM to balance key demographic and clinical covariates, and the results remained consistent in sensitivity analyses.

Future research should focus on conducting prospective cohort studies to further explore the relationship between cancer treatment and changes in renal function. Larger sample sizes and the inclusion of more diverse patient populations from multiple centers will help validate our findings and enhance the external validity of the results.

## Conclusion

Cancer patients exhibited a higher baseline eGFR compared with non-cancer individuals, followed by a more pronounced decline in renal function over time. The initially elevated eGFR may represent a transient, cancer-related hyperfiltration pattern reflecting adaptive renal changes to metabolic or hemodynamic stress rather than true preservation of kidney health. After treatment initiation, this apparent functional advantage diminished, with cancer patients showing a faster eGFR decline, potentially influenced by treatment exposure and pre-existing hyperfiltration vulnerability. While these observations are descriptive and do not imply causality, they underscore the importance of early and continuous renal function monitoring in cancer patients to guide individualized treatment and prevent long-term kidney impairment.

## Data Availability

The raw data supporting the conclusions of this article will be made available by the authors, without undue reservation.

## Supplementary material

FIGURE S1 Comparison of eGFR, Renal Parenchymal Volume, and Annual GFR Decline between Cancer and Non-Cancer Groups at Other Centers. **(A)** eGFR in cancer patients compared to non-cancer individuals by age at center 2. **(B–D)** eGFR in cancer vs. non-cancer groups stratified by different factors at center 2: **(B)** by age and gender, **(C)** by comorbidity, and **(D)** by cancer stage. **(E)** eGFR in cancer patients compared to non-cancer individuals by age at center 3. **(F–H)** eGFR in cancer vs. non-cancer groups stratified by different factors at center 3: **(F)** by age and gender, **(G)** by comorbidity, and **(H)** by cancer stage. Data for the non-cancer groups are derived from a combined dataset of health examination records from center 5 and center 6. Statistical significance is indicated by asterisks (*), with **p* < 0.05, ***p* < 0.01, and ****p* < 0.001. Colors denote specific group comparisons: **(A/E)** cancer vs. non-cancer by age; **(B/F)** blue for male and red for female (cancer vs. non-cancer); **(C/G)** blue for no comorbidity and red for comorbidity (cancer vs. non-cancer); **(D/H)** comparisons between each cancer stage and the non-cancer group, with asterisk colors matching the respective cancer stages.

## References

[1] Siegel RL Giaquinto AN Jemal A. Cancer statistics, 2024. CA Cancer J Clin. (2024) 74:12–49. doi: 10.3322/caac.21820, PMID: 38230766

[2] Christiansen CF Johansen MB Langeberg WJ Fryzek JP Sorensen HT. Incidence of acute kidney injury in cancer patients: a Danish population-based cohort study. Eur J Intern Med. (2011) 22:399–406. doi: 10.1016/j.ejim.2011.05.005 21767759

[3] Tonelli M Lloyd A Cheung WY Hemmelgarn BR James MT Ravani P . Mortality and resource use among individuals with chronic kidney disease or cancer in Alberta, Canada, 2004-2015. JAMA Netw Open. (2022) 5:e2144713. doi: 10.1001/jamanetworkopen.2021.44713, PMID: 35076702 PMC8790674

[4] Kitchlu A Reid J Jeyakumar N Dixon SN Munoz AM Silver SA . Cancer risk and mortality in patients with kidney disease: a population-based cohort study. Am J Kidney Dis. (2022) 80:436–448.e1. doi: 10.1053/j.ajkd.2022.02.020, PMID: 35405208

[5] Hong J Lee S Chun G Jung JY Park J Ahn JY . Baseline renal function as a prognostic indicator in patients with newly diagnosed diffuse large B-cell lymphoma. Blood Res. (2016) 51:113–21. doi: 10.5045/br.2016.51.2.113, PMID: 27382556 PMC4931929

[6] Launay-Vacher V Oudard S Janus N Gligorov J Pourrat X Rixe O . Prevalence of renal insufficiency in cancer patients and implications for anticancer drug management: the renal insufficiency and anticancer medications (IRMA) study. Cancer. (2007) 110:1376–84. doi: 10.1002/cncr.22904 17634949

[7] Canter D Viterbo R Kutikov A Wong YN Plimack E Zhu F . Baseline renal function status limits patient eligibility to receive perioperative chemotherapy for invasive bladder cancer and is minimally affected by radical cystectomy. Urology. (2011) 77:160–5. doi: 10.1016/j.urology.2010.03.091, PMID: 20709369

[8] Malyszko J Lee MW Capasso G Kulicki P Matuszkiewicz-Rowinska J Ronco P . How to assess kidney function in oncology patients. Kidney Int. (2020) 97:894–903. doi: 10.1016/j.kint.2019.12.023, PMID: 32229094

[9] Claudel SE Gandhi M Patel AB Verma A. Estimating kidney function in patients with cancer: a narrative review. Acta Physiol (Oxf). (2023) 238:e13977. doi: 10.1111/apha.13977, PMID: 37057998 PMC11839183

[10] Jin J Wang Y Shen Q Gong J Zhao L He Q. Acute kidney injury in cancer patients: a nationwide survey in China. Sci Rep. (2019) 9:3540. doi: 10.1038/s41598-019-39735-9, PMID: 30837515 PMC6401015

[11] Salahudeen AK Doshi SM Pawar T Nowshad G Lahoti A Shah P. Incidence rate, clinical correlates, and outcomes of AKI in patients admitted to a comprehensive cancer center. Clin J Am Soc Nephrol. (2013) 8:347–54. doi: 10.2215/CJN.03530412, PMID: 23243268 PMC3586962

[12] Konigsbrugge O Lotsch F Zielinski C Pabinger I Ay C. Chronic kidney disease in patients with cancer and its association with occurrence of venous thromboembolism and mortality. Thromb Res. (2014) 134:44–9. doi: 10.1016/j.thromres.2014.04.002, PMID: 24792954

[13] Ciorcan M Chisavu L Mihaescu A Gadalean F Bob FR Negru S . Chronic kidney disease in cancer patients, the analysis of a large oncology database from Eastern Europe. PLoS One. (2022) 17:e0265930. doi: 10.1371/journal.pone.0265930, PMID: 35679539 PMC9183451

[14] Finetti F Travelli C Ercoli J Colombo G Buoso E Trabalzini L. Prostaglandin E2 and cancer: insight into tumor progression and immunity. Biology (Basel). (2020) 9:434. doi: 10.3390/biology9120434, PMID: 33271839 PMC7760298

[15] Audoly LP Ruan X Wagner VA Goulet JL Tilley SL Koller BH . Role of EP(2) and EP(3) PGE(2) receptors in control of murine renal hemodynamics. Am J Physiol Heart Circ Physiol. (2001) 280:H327–33. doi: 10.1152/ajpheart.2001.280.1.H327 11123248

[16] Vakkala M Paakko P Soini Y. eNOS expression is associated with the estrogen and progesterone receptor status in invasive breast carcinoma. Int J Oncol. (2000) 17:667–71. doi: 10.3892/ijo.17.4.667, PMID: 10995876

[17] Cobbs CS Brenman JE Aldape KD Bredt DS Israel MA. Expression of nitric oxide synthase in human central nervous system tumors. Cancer Res. (1995) 55:727–30. 7531613

[18] Xu W Liu LZ Loizidou M Ahmed M Charles IG. The role of nitric oxide in cancer. Cell Res. (2002) 12:311–20. doi: 10.1038/sj.cr.7290133, PMID: 12528889

[19] Vadi S Yim K. Hypernatremia due to urea-induced osmotic diuresis: physiology at the bedside. Indian J Crit Care Med. (2018) 22:664–9. doi: 10.4103/ijccm.IJCCM_266_18, PMID: 30294134 PMC6161575

[20] Davison AM. Renal diseases associated with malignancies. Nephrol Dial Transplant. (2001) 16:13–4. doi: 10.1093/ndt/16.suppl_6.13, PMID: 11568228

