# Supplementary figures and images for: Comparative study of eGFR in cancer and non-cancer individuals: a multicenter analysis
Source: Front Med (Lausanne). 2025 Dec 4;12:1642162. doi: 10.3389/fmed.2025.1642162 (PMC12711550; doi:10.3389/fmed.2025.1642162)

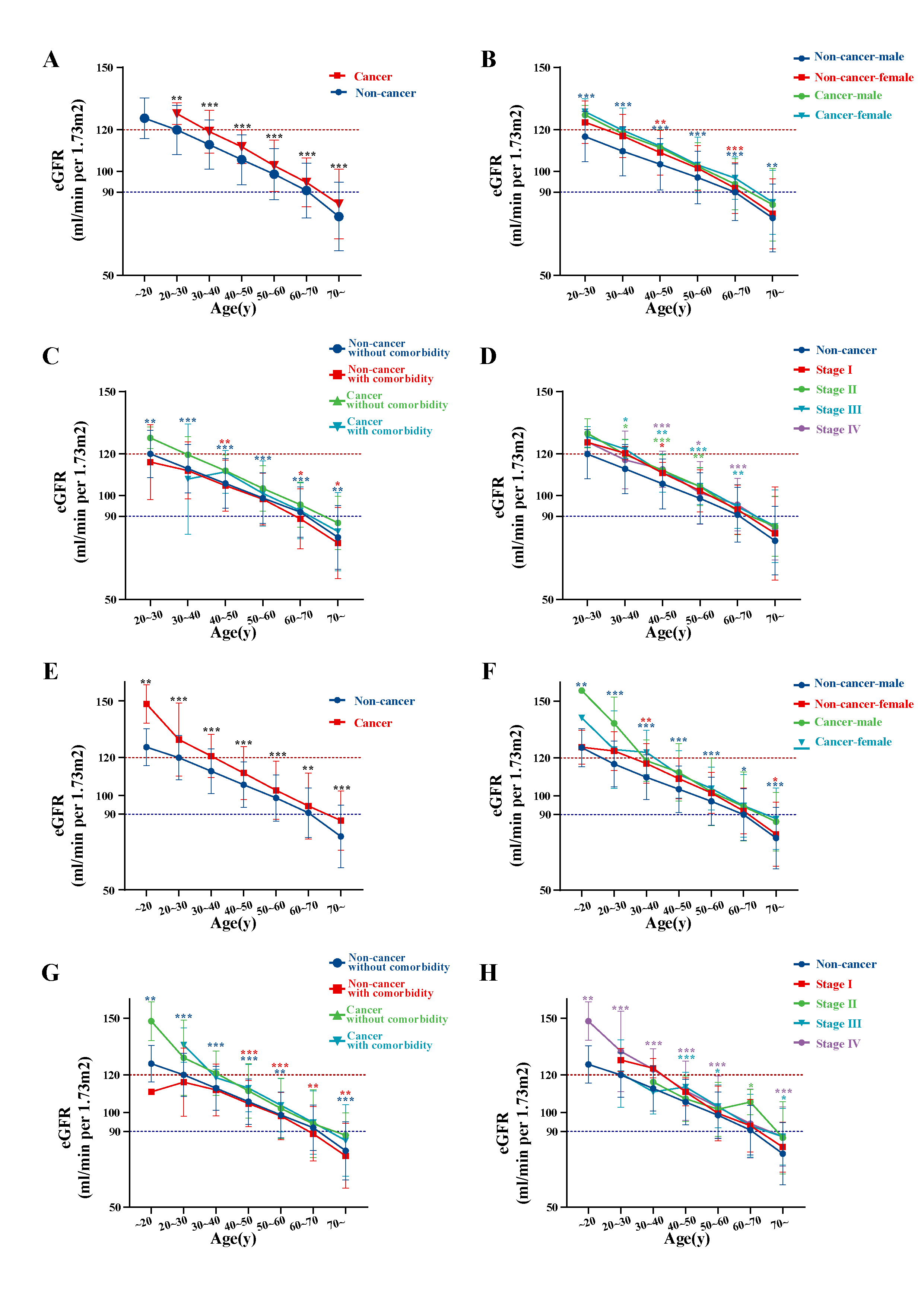

Supplement: FIGURE S1 — Comparison of eGFR, Renal Parenchymal Volume, and Annual GFR Decline between Cancer and Non-Cancer Groups at Other Centers. (A) eGFR in cancer patients compared to non-cancer individuals by age at center 2. (B–D) eGFR in cancer vs. non-cancer groups stratified by different factors at center 2: (B) by age and gender, (C) by comorbidity, and (D) by cancer stage. (E) eGFR in cancer patients compared to non-cancer individuals by age at center 3. (F–H) eGFR in cancer vs. non-cancer groups stratified by different factors at center 3: (F) by age and gender, (G) by comorbidity, and (H) by cancer stage. Data for the non-cancer groups are derived from a combined dataset of health examination records from center 5 and center 6. Statistical significance is indicated by asterisks (*), with *p < 0.05, **p < 0.01, and ***p < 0.001. Colors denote specific group comparisons: (A/E) cancer vs. non-cancer by age; (B/F) blue for male and red for female (cancer vs. non-cancer); (C/G) blue for no comorbidity and red for comorbidity (cancer vs. non-cancer); (D/H) comparisons between each cancer stage and the non-cancer group, with asterisk colors matching the respective cancer stages. [file Image_1.tif]
