# Supplementary material for: Comparative study of eGFR in cancer and non-cancer individuals: a multicenter analysis
Source: Front Med (Lausanne). 2025 Dec 4;12:1642162. doi: 10.3389/fmed.2025.1642162 (PMC12711550; doi:10.3389/fmed.2025.1642162)
Supplement: Supplementary file 2 [file Table_1.docx]

**Table S1. Baseline Characteristics Before Propensity Score Matching**

| **Variable** | **Overall**  **(n = 51,523)** | **Non-Cancer**  **(n = 27,045)** | **Cancer**  **(n = 24,478)** | **p-value** | **SMD** |
| --- | --- | --- | --- | --- | --- |
| **Sex (%)** |  |  |  |  |  |
| Woman | 26,390 (51.2%) | 14,885 (55.0%) | 11,505 (47.0%) | <0.001 | 0.161 |
| Man | 25,133 (48.8%) | 12,160 (45.0%) | 12,973 (53.0%) |  |  |
| **Age (mean± SD)** | 48.72 (14.89) | 40.98 (±12.20) | 57.27 (±12.77) | <0.001 | 1.305 |
| **BMI (mean ± SD)** | 22.85 (3.34) | 23.09 (±3.30) | 22.57 (±3.35) | <0.001 | 0.157 |
| **Diabetes (DM) (%)** |  |  |  |  |  |
| No | 48,273 (93.7%) | 25,907 (95.8%) | 22,366 (91.4%) | <0.001 | 0.181 |
| Yes | 3,250 (6.3%) | 1,138 (4.2%) | 2,112 (8.6%) |  |  |
| **Hypertension (HTN) (%)** |  |  |  |  |  |
| No | 45,692 (88.7%) | 24,129 (89.2%) | 21,563 (88.1%) | <0.001 | 0.036 |
| Yes | 5,831 (11.3%) | 2,916 (10.8%) | 2,915 (11.9%) |  |  |

**Abbreviations:** SD**,** standard deviation; BMI, body mass index; SMD, standard mean difference.
