# Supplementary material for: Comparative study of eGFR in cancer and non-cancer individuals: a multicenter analysis
Source: Front Med (Lausanne). 2025 Dec 4;12:1642162. doi: 10.3389/fmed.2025.1642162 (PMC12711550; doi:10.3389/fmed.2025.1642162)
Supplement: Supplementary file 3 [file Table_2.docx]

**Table S2. Multivariable Linear Regression Analysis for Baseline eGFR in the Matched Cohort**

| **Variable** | **β** | **Standard Error** | **t-value** | **95% CI** | ***p*-value** |
| --- | --- | --- | --- | --- | --- |
| Cancer group (vs. non-cancer) | 8.56 | 0.14 | 60.61 | 8.29 – 8.84 | <0.001 |
| Age (per year) | –0.82 | 0.006 | –126.84 | –0.83 – –0.81 | <0.001 |
