# Supplementary material for: Comparative study of eGFR in cancer and non-cancer individuals: a multicenter analysis
Source: Front Med (Lausanne). 2025 Dec 4;12:1642162. doi: 10.3389/fmed.2025.1642162 (PMC12711550; doi:10.3389/fmed.2025.1642162)
Supplement: Supplementary file 4 [file Table_3.docx]

**Table S3. Baseline Characteristics Before Propensity Score Matching**

| **Variable** | **Overall (n = 1,055)** | **Non-Cancer  (n = 487)** | **Cancer  (n = 568)** | **P** | **SMD** |
| --- | --- | --- | --- | --- | --- |
| **Sex (%)** |  |  |  |  |  |
| Woman | 0.63 (0.48) | 0.63 (0.48) | 0.62 (0.49) | 0.758 | 0.019 |
| Man | 0.37 (0.48) | 0.37 (0.48) | 0.38 (0.49) |  |  |
| **Age (mean ± SD)** | 47.80 (±11.34) | 47.32 (±12.11) | 48.22 (±10.62) | 0.2 | 0.079 |
| **BMI (mean ± SD)** | 23.35 (±3.47) | 24.20 (±3.28) | 22.62 (±3.46) | <0.001 | 0.469 |
| **Diabetes (DM) (%)** |  |  |  |  |  |
| No | 0.06 (0.24) | 0.07 (0.25) | 0.05 (0.23) | 0.447 | 0.047 |
| Yes | 0.06 (0.24) | 0.07 (0.25) | 0.05 (0.23) |  |  |
| **Hypertension (HTN) (%)** |  |  |  |  |  |
| No | 0.08 (0.28) | 0.12 (0.32) | 0.06 (0.23) | <0.001 | 0.217 |
| Yes | 0.08 (0.28) | 0.12 (0.32) | 0.06 (0.23) |  |  |
| **Kidney Volume  (mean ± SD)** | 276 (±53) | 27 (±52) | 280 (±54) | 0.008 | 0.163 |

**Abbreviations:** SD**,** standard deviation; BMI, body mass index; SMD, standard mean difference.
